# Cardiomiopatia de Takotsubo Recorrente: Um Enigma ainda não Resolvido

**DOI:** 10.36660/abc.20200140

**Published:** 2020-09-18

**Authors:** Kenan Yalta, Caglar Kaya

**Affiliations:** 1 Trakya University School of Medicine Cardiology Department Edime Turquia Trakya University, School of Medicine, Cardiology Department,Edime - Turquia

**Keywords:** Síndrome de Takotsubo, Cardiomiopatia de Takotsubo, Recidiva, Revisão Sistemática

Nos últimos anos, a Cardiomiopatia de Takotsubo (CTT) ganhou um amplo reconhecimento como uma forma transitória de disfunção miocárdica, geralmente surgindo em resposta a certos fatores desencadeantes de estresse associados à descarga adrenérgica, e foi relatado em estudos anteriores que o mesmo tem taxas de recorrência variáveis.^1-7^ Em sua revisão sistemática^1^ recentemente publicada, Campos et al.^1^ sugeriram certos fatores associados à recorrência da CTT, incluindo índice de massa corporal (IMC) mais baixo, gênero feminino, gradiente médio-ventricular (GMV) já existente e proximidade temporal com o evento índice (inicial) da CTT. Concordamos plenamente com esses fatores de risco específicos neste cenário. No entanto, gostaríamos de ter mais informações sobre sua análise e fazer alguns comentários no contexto geral de recorrência da CTT. Como descrito abaixo, acreditamos que existam duas categorias básicas de pacientes com CTT que são mais propensos a ter recorrências futuras na prática clínica:

- A primeira categoria compreende os casos de CTT associados a um gatilho puramente mecânico, incluindo o GMV.^1^ Essa forma de evolução da CTT foi encontrada principalmente no contexto de cardiomiopatia hipertrófica (CMH) e doença cardíaca hipertensiva, geralmente com uma pequena cavidade ventricular (mesmo sem GMV evidente em repouso), e parece ser desencadeada por aumentos súbitos e excessivos no GMV, levando a um padrão de balonismo apical,^2,3,6^ geralmente de forma recorrente.^1^ Portanto, a reconstrução cirúrgica do médio-ventrículo pode ser obrigatória em certos casos refratários com surtos recorrentes de CTT.^6^ Assim, questionamos a incidência de CMH e doença cardíaca hipertensiva na análise geral. E em relação à reconstrução cirúrgica, se houver, e seu impacto na recorrência da CTT? Digno de nota, o maior risco de recorrência da CTT no sexo feminino^1^pode, até certo ponto, ser atribuível à maior incidência de GMV sutil ou evidente, em sua maior parte associada a dimensões cavitárias relativamente pequenas e incidência relativamente maior de hipertrofia miocárdica significativa (em resposta à hipertensão sistêmica, etc.) em mulheres.
- A segunda categoria constitui a parte principal e pode incluir aqueles com uma descarga adrenérgica grave durante o evento índice de CTT.^4,6,7^ É importante ressaltar que a descarga adrenérgica grave é bem conhecida por estar significativamente associada a distúrbios inerentes^6^ que levam a mecanismos de resposta fisiológica exagerados (ao invés do grau do gatilho associado), potencialmente sugerindo sua natureza repetitiva e facilmente induzível em um determinado caso de CTT. A proximidade temporal com ao evento índice de CTT, sugerido como um fator de risco na análise atual,^1^ também pode substanciar o papel da descarga adrenérgica grave em futuras recorrências de CTT. Mais especificamente, a descarga adrenérgica grave, além de ser determinada diretamente com os níveis plasmáticos de catecolaminas, pode potencialmente apresentar uma variedade de sinais específicos, embora indiretos, incluindo gradiente agudo da via de saída do ventrículo esquerdo (VSVE) (na imagem cardíaca) e padrão de fluxo coronariano lento (FCL) (na angiografia coronária invasiva) em pacientes com CTT.^4-7^ Consequentemente, os casos com e sem recorrência de CTT diferiram significativamente em relação a esses sinais específicos durante seus eventos índice? Esses sinais^4,6,7^ também podem servir como potenciais preditores para recorrências de CTT? E quanto ao impacto das opções terapêuticas radicais, incluindo o bloqueio^2,6,7^ do gânglio simpático, se houver, nas recorrências de CTT?

Em suma, os autores^1^ devem ser parabenizados pela análise bem executada. Entretanto, mais estudos ainda são necessários para estabelecer completamente os preditores absolutos de recorrências futuras de CTT (possivelmente com a sugestão de um escore de risco simples), juntamente com estratégias de manejo específicas para a prevenção dessas recorrências.

## References

[1] 1. Campos FAD, Ritt LEF, Costa JPS, Cruz CM, Feitosa-Filho GS, Oliveira QB, et al. Factors Associated with Recurrence in Takotsubo Syndrome: A Systematic Review. Arq Bras Cardiol. 2020;114(3):477-83. 10.36660/abc.20180377 PMC7792734 32049155

[2] 2. Sherid M, Riedy K, Rosenzweig B, Massera D, Saric M, Swistel DG, et al. Distinctive Hypertrophic Cardiomyopathy Anatomy and Obstructive Physiology in Patients Admitted with Takotsubo Syndrome. Am J Cardiol. 2020; 125(11):1700-9. 10.1016/j.amjcard.2020.02.013 32278461

[3] 3. Vinardell JM, Mihos CG, Nader A, Ro R, Escolar E, Santana O. Stress Cardiomyopathy in a Patient with Hypertrophic Cardiomyopathy: Case Presentation and Review of the Literature. Rev Cardiovasc Med. 2018; 19(2): 65—8. 10.31083/j.rcm.2018.02.893 31032604

[4] 4. Kishida K, Woo E, Sasaki T, Hamori K, Daimon M, Mieno S, et al. Left Ventriculoplasty in a Patient With Suspected Takotsubo Cardiomyopathy Followed by a Left Ventricular Aneurysm. *Kyobu Geka*. 2014;67(5):419-22. 24917292

[5] 5. Elliott PM, Anastasakis A, Borger MA, Borggrefe M, Cecchi F, Charron P, et al. 2014 ESC Guidelines on diagnosis and management of hypertrophic cardiomyopathy: the Task Force for the Diagnosis and Management of Hypertrophic Cardiomyopathy of the European Society of Cardiology (ESC). *Eur Heart J*. 2014;35(39):2733-79. 10.1093/eurheartj/ehu284 25173338

[6] 6. Verschure DO, Somsen GA, van Eck-Smit BL, Knol RJ, Booij J, Verberne HJ. Tako-tsubo cardiomyopathy: how to understand possible pathophysiological mechanism and the role of (123)I-MIBG imaging. *J Nucl Cardiol*. 2014;21(4):730-8. 10.1007/s12350-014-9855-y 24464623

[7] 7. Akashi YJ, Nakazawa K, Sakakibara M, Miyake F, Musha H, Sasaka K. 123I-MIBG myocardial scintigraphy in patients with “takotsubo” cardiomyopathy. *J Nucl Med*. 2004;45(7):1121-7. 15235057

