# Supplementary material for: Cardiomiopatia de Takotsubo Recorrente: Um Enigma ainda não Resolvido
Source: Arq Bras Cardiol. 2020 Sep 18;115(3):590–1. [Article in Portuguese] doi: 10.36660/abc.20200140 (PMC9363086; doi:10.36660/abc.20200140)
Supplement: Translation

# Translation: Recurrent Takotsubo Cardiomyopathy: A Puzzle Yet to be Solved

**Authors:** Kenan Yalta, Caglar Kaya

**Affiliations:** 1 Turkey Trakya University, School of Medicine, Cardiology Department, Edime - Turkey

**Keywords:** Takotsubo Syndrome, Takotsubo Cardiomyopathy, Recurrence, Systematic Review

In the past years, takotsubo cardiomyopathy (TTC) has gained widespread recognition as a transient form of myocardial dysfunction, usually arising in response to certain stressful triggers Trakya University, School of Medicine, Cardiology Department,1 Edime - Turkey adrenergic discharge, and has been reported to have variable recurrence rates in previous studies.^1-7^ In their recently published systematic review,^1^ Campos et al.^1^ have suggested certain factors associated with TTC recurrence, including lower body mass index (BMI), female gender, an existing mid-ventricular gradient (MVG) and temporal proximity to the index (initial) TTC event. We fully agree on these particular risk factors in this setting. However, we would like to have further information regarding their analysis and make a few comments in the general context of TTC recurrence. As described below, we hold the opinion that there are two basic categories of TTC patients who are more likely to have future recurrences in clinical practice:

- First category: comprises TTC cases associated with a purely mechanical trigger including MVG.^1^ This form of TTC evolution has been mostly encountered in the setting of hypertrophic cardiomyopathy (HCM) and hypertensive heart disease, usually with a small ventricular cavity (even without an overt MVG at rest), and appears to be triggered by sudden and excessive increases in MVG leading to an apical ballooning pattern,^2,3,6^ generally in a recurrent fashion.^1^ Therefore, surgical reconstruction of the mid-ventricle might be mandatory in certain refractory cases with recurrent TTC bouts.^6^ Accordingly, we wonder about the incidences of HCM and hypertensive heart disease in the overall analysis. What about surgical reconstruction, if any, and its impact on TTC recurrence? Of note, the higher risk of TTC recurrence in female gender^1^ might, to some extent, be attributable to the higher incidence of subtle or overt MVG, mostly associated with relatively small cavity dimensions and relatively higher incidence of significant myocardial hypertrophy (in response to systemic hypertension, etc.) in women.
- Second category constitutes the major portion and might include those with a severe adrenergic discharge during their index TTC event.^4,6,7^ Importantly, severe adrenergic discharge is well^6^ known to be significantly associated with inherent disorders leading to exaggerated physiological response mechanisms (rather than the degree of the associated trigger), potentially suggesting its repetitive and easily inducible nature in a given TTC case. Temporal proximity to the index TTC, suggested as a risk factor in the current analysis,^1^ also substantiates the role of severe adrenergic discharge in future TTC recurrences. More specifically, severe adrenergic discharge, besides being directly determined by plasma catecholamine levels, might potentially present with a variety of specific, yet indirect signs, including acute left ventricular outflow tract (LVOT) gradient (on cardiac imaging) and coronary slow flow (CSF) pattern (on invasive coronary angiogram) in patients with TTC.^4-7^ Accordingly, did cases with and without a TTC recurrence significantly differ in terms of these specific signs during their index events? Might these signs^4,6,7^ also serve as potential predictors of TTC recurrences? What about the impact of radical therapeutic options, including sympathetic ganglion blockade,^2,6,7^ if any, on TTC recurrences?

In summary, the authors1 should be congratulated for their well-performed analysis. However, further studies are still warranted to fully establish absolute predictors of future TTC recurrences (possibly with the suggestion of a simple risk score) along with specific management strategies for the prevention of these recurrences.
