# Supplementary material for: Cardiomiopatia de Takotsubo Recorrente: Um Enigma ainda não Resolvido
Source: Arq Bras Cardiol. 2020 Sep 18;115(3):590–1. [Article in Portuguese] doi: 10.36660/abc.20200140 (PMC9363086; doi:10.36660/abc.20200140)
Supplement: Related texts

# Letter: Carta-Resposta

**Authors:** Luiz Eduardo Fonteles Ritt

**Affiliations:** 1 Escola Bahiana de Medicina e Saúde Pública Centro de Estudos Clínicos Hospital Cardio Pulmonar Brasil Hospital Cardio Pulmonar - Centro de Estudos Clínicos Escola Bahiana de Medicina e Saúde Pública

Primeiramente, agradecemos aos autores os comentários a respeito de nosso recente artigo nos Arquivos Brasileiros de Cardiologia intitulado “Fatores Associados à Recorrência na Síndrome de Takotsubo: Uma Revisão Sistemática”.^1^ Na carta, os autores fizeram alguns comentários adicionais interessantes sobre os mecanismos de recorrência de Takotsubo relacionados a gatilhos mecânicos associados ao gradiente médio-ventricular que podem estar relacionados à cardiomiopatia hipertrófica (CMH) associada e a um segundo mecanismo relacionado à descarga adrenérgica grave.

Uma questão que eles colocaram estava relacionada às incidências de CMH e cardiopatia hipertensiva nos dados que analisamos. Na verdade, nos artigos originais que foram revisados, os autores não mencionaram as incidências de CMH ou cardiopatia hipertensiva. Alguns pacientes com Síndrome de Takotsubo (ST) podem apresentar movimento anterior sistólico da válvula mitral e obstrução ventricular, sendo que às vezes é difícil determinar se essas características estão relacionadas a ST ou CMH associada ou mesmo sua combinação e o acompanhamento clínico, eletrocardiográfico e de imagem pode revelar o diagnóstico.^2,3^ Sobre o impacto da reconstrução cirúrgica na recorrência, em uma breve revisão da literatura encontramos um artigo que descreve um caso de reconstrução ventricular em uma mulher que permaneceu com aneurisma apical e eventos embólicos recorrentes.^4^ Dessa forma, no caso de obstrução permanente da via de saída ventricular em pacientes com CMH que apresentavam ST pode-se considerar a indicação das diretrizes da CMH para intervenção cirúrgica.^5^

Em relação à descarga adrenérgica, concordamos que esta pode ser outra característica a ser lembrada em estudos futuros para melhor analisar sua relação com a gravidade e recorrência da ST. Estudos utilizando cintilografia miocárdica com 123I-MIBG demonstraram uma diminuição de captação na região com contratilidade ventricular comprometida e a persistência desse comprometimento estaria relacionada a maior chance de recorrência.^6,7^ Sobre o bloqueio ganglionar simpático, entendemos que pode ser um campo de pesquisa para pacientes com vários casos de recorrência; nenhum estudo foi encontrado em uma busca booleana no Pubmed com os termos bloqueio simpático ou bloqueio ganglionar ou bloqueio ganglionar cirúrgico e Takotsubo.

Novas pesquisas serão valiosas para uma melhor compreensão do prognóstico e das opções de tratamento para as síndromes de Takotsubo.

# Letter: Reply

**Authors:** Luiz Eduardo Fonteles Ritt

**Affiliations:** 1 Escola Bahiana de Medicina e Saúde Pública Centro de Estudos Clínicos Hospital Cardio Pulmonar Brasil Hospital Cardio Pulmonar - Centro de Estudos Clínicos Escola Bahiana de Medicina e Saúde Pública

First, we thank the authors for the comments regarding our recent article in *Arquivos Brasileiros de Cardiologia* entitled “Factors Associated with Recurrence in Takotsubo Syndrome: A Systematic Review”.^1^ In the letter, the authors made some interesting additional comments regarding the mechanisms of Takotsubo recurrence related to mechanical triggers related to mid-ventricular gradient that may be related to associated hypertrophic cardiomyopathy (HCM) and a second mechanism related to severe adrenergic discharge.

One question they posed was related to the incidence of HCM and hypertensive heart disease in the data we analyzed. Actually, in the original papers that were revised, the authors did not mention HCM or hypertensive heart disease incidence. Some patients with Takotsubo Syndrome (TS) may present with systolic anterior motion of the mitral valve and ventricular obstruction; it is sometimes difficult to determine whether these features are related to TS or associated to HCM or even their combination and the clinic, electrocardiographic and image follow-up may reveal the diagnosis.^2,3^ About the impact of the surgical reconstruction on recurrence, in a brief review of literature we found one article describing a case of ventricular reconstruction in a female who remained with apical aneurism and recurrent embolic events.^4^ Therefore, in the case of permanent ventricular outflow obstruction in HCM who has TS, one may consider the indication of the HCM guidelines for surgical intervention.^5^

Regarding the adrenergic discharge, we agree that this may be another characteristic to be kept in mind for future studies to better analyze its association with TS severity and recurrence. Studies using 123I-MIBG myocardial scintigraphy have shown decreased uptake in the ventricular contractility-impaired region and the persistence of this impairment would be related to a higher chance of recurrence.^6,7^ About the sympathetic ganglion blockade, we understand that it may be a field for research in patients with several cases of recurrences; no studies were found in a Boolean search in Pubmed using the terms sympathetic blockade or ganglion blockade or surgical ganglion blockade and takotsubo.

Further studies will be invaluable for a better understanding of prognosis and treatment options for Takotsubo Syndrome.
